# Production of a Potentially Probiotic Product for Animal Feed and Evaluation of Some of Its Probiotic Properties

**DOI:** 10.3390/ijms221810004

**Published:** 2021-09-16

**Authors:** Rubén Agregán-Pérez, Elisa Alonso-González, Juan Carlos Mejuto, Nelson Pérez-Guerra

**Affiliations:** 1 Department of Analytical and Food Chemistry, Faculty of Sciences, Ourense Campus, University of Vigo, As Lagoas s/n, 32004 Ourense, Spain; rubenag85@gmail.com (R.A.-P.); elisa.alonso@uvigo.es (E.A.-G.); 2 Department of Physical Chemistry, Faculty of Sciences, Ourense Campus, University of Vigo, As Lagoas s/n, 32004 Ourense, Spain

**Keywords:** fed-batch fermentation, kefir grains, probiotic culture, whey

## Abstract

Nowadays, probiotics have been proposed for substituting antibiotics in animal feed since the European Union banned the latter compounds in 2006 to avoid serious side effects on human health. Therefore, this work aimed to produce a probiotic product for use in animal feed by fed-batch fermentation of whey with a combination of kefir grains, AGK1, and the fermented whole milk used to activate these kefir grains. The probiotic culture obtained was characterized by high levels of biomass (8.03 g/L), total viability (3.6 × 10^8^ CFU/mL) and antibacterial activity (28.26 Activity Units/mL). Some probiotic properties of the probiotic culture were investigated in vitro, including its survival at low pH values, under simulated gastrointestinal conditions, after freezing in skim milk at −20 °C, and in the commercial feed during storage at room temperature. The viable cells of lactic and acetic acid bacteria and yeasts exhibited higher tolerance to acidic pH and simulated gastrointestinal conditions when the cells were protected with skim milk and piglet feed, compared with washed cells. The results indicated the feasibility of producing a probiotic product at a low cost with a potential application in animal feed.

## 1. Introduction

Nowadays, there is growing scientific and commercial interest in the use of probiotics in animal feed to prevent or treat different animal diseases [1] as an alternative to the use of growth promoter antibiotics, which could produce adverse reactions and side effects on the animals [2]. According to the joint Food and Agriculture Organization of the United Nations (FAO) and World Health Organization (WHO) Working Group, these probiotics are defined as living microorganisms, which when administered in adequate amounts, promote a health benefit on the host [1,2,3]. The consumption of these microorganisms contributes to the establishment of an intestinal population beneficial for the host and antagonist for disease-causing bacteria, which could contribute to improving the efficiency in animal production systems [1,2].

Since probiotic effects are species- and strain-specific, after formulating a probiotic product, strains should be characterized using phenotypic and genotypic methods, and the functional and safety aspects of these microorganisms in feed should be examined [4,5].

For their use as probiotics, the candidate microorganisms should have the following characteristics: (i) nonpathogenic, nontoxic and noncarcinogenic; (ii) resistant to gastrointestinal transit conditions; (iii) able to adhere to host epithelial tissue and reduce the adhesion of pathogens; (iv) amenable to be produced at industrial scale; (v) able to produce higher concentrations of antimicrobial substances (e.g., organic acids and bacteriocins) with activity against pathogenic bacteria; (vi) beneficial to the host animal in some way; (vii) able to persist within the gastrointestinal tract, among others [4].

The most commonly used probiotics are some strains of lactic acid bacteria (LAB), *Bacillus* and yeasts [2,6], which have been formulated in many types of animal diets. However, when combined, probiotics could produce a higher positive effect on efficiency in animal production systems [7].

In this way, the use of kefir grains as a probiotic for animal feeds would be advantageous considering the complex microbiological composition of the grains and the simplicity of its production both at industrial and artisanal scale. Thus, the most common bacterial strains identified in the kefir grains are LAB, including *Lactobacillus* (*Lb*.) *acidophilus*, *Lb*. *casei*, *Lb*. *helveticus*, *Lb*. *bulgaricus*, *Lb*. *parakefir*, *Lb*. *kefiranofaciens*, *Lb*. *kefirgranum*, *Lb*. *kefir*, *Lb*. *plantarum*, *Lb*. *delbrueckii* subsp. *bulgaricus*, *Lb*. *rhamnosus*, *Lb*. *fructivorous*, *Lb*. *hilgardii*, *Lb*. *paracasei*, *Lb*. *fermentum*, *Lb*. *crispatus*, *Lb*. *gallinarum*, *Lb*. *reuteri*, *Lb*. *brevis*, *Bifidobacterium bifidum*, *Leuconostoc mesenteroids* subsp. *cremoris*, *Streptococcus thermophilus*, *Lactococcus lactis*, *Enterococcus durans*, *Pediococcus* (*Ped*.) *acidilactici*, *Ped*. *dextrinicus*, *Ped*. *pentosaceus*, and acetic acid bacteria, including *Acetobacter* (*A*.) *aceti*, *A*. *lovaniensis* and *A*. *syzgii* [8,9]. Although fewer studies have been conducted regarding the characterization of the yeasts population of kefir grains, the following species have been identified: *Zygosaccharomyces* (*Z*.) sp., *Z*. *rouxii*, *Candida* (*C*.) *lipolytica*, *C*. *holmii*, *C*. *inconspicua*, *C*. *maris*, *C*. *kefyr*, *C*. *lambica*, *C*. *krusei*, *Kluyveromyces* (*K*.) *marxianus*, *K*. *lactis*, *Saccharomyces* (*S*.) *cerevisiae*, *S*. *fragilis*, *S*. *lactis*, *S*. *lipolytica*, *Torulaspora* (*T*.) *delbrus*, *T*. *delbrueckii*, *Debaryomyces hansenii*, *Kazachstania aerobia*, *Lachancea meyersii* and *Geotrichum candidum* [9].

In addition to the demonstrated probiotic properties of many of the above-mentioned bacterial and yeast strains [10], different species of *Lactobacillus*, including *Lb*. *kefiranofaciens*, *Lb*. *kefirgranum*, *Lb*. *parakefir*, *Lb*. *kefir* and *Lb*. *delbrueckii* subsp. *bulgaricus* can produce kefiran [11]. This edible, biodegradable and safe water-soluble exopolysaccharide has been used in medicine due to its antimicrobial, antitumor, anti-inflammatory, antioxidant, biocidal and hypotensive activities. Other properties have been attributed to kefiran, including its capability for the modulation of the gut immune system, reduction of high blood pressure (hypertension) and protection of epithelial cells against microbial toxins [11].

However, for the production of a probiotic product, the use of cheap substrates (mainly some wastes from the food industry, such as whey) and an appropriate fermentation procedure is needed to reduce the production cost.

The use of whey as a fermentation substrate could be advantageous because this offers the possibility of recycling this food waste in the production of a probiotic product for animal feed, avoiding the disposal of whey and consequently reducing its impact on the environment [12]. In addition, the kefir grains, used as a fermentation entity, provide a complex microbiota that could enhance the probiotic properties of the fermented probiotic product, as observed by Zhang et al. [7].

Although in some studies [13,14,15], whey has been fermented with kefir grains, there is no information available on the kinetics of important culture variables including pH, and the concentrations of biomass, viable cells, sugars, proteins and products (e.g., antibacterial activity, organic acids and ethanol).

The inoculation of whey with a combination of kefir grains and free biomass from fermented milk used in the activation of the kefir grains could be advantageous when considering a large-scale industrial production of the probiotic product. With this approach, larger volumes of whey could be inoculated compared to the volumes that could be inoculated only with kefir grains. This is mainly because the inoculation of large volumes of whey with the latter inoculum would require the generation of a large mass of kefir grains, whose activation in several subcultures (passages) would lead to excessive milk consumption, increasing the production cost of the fermentation process.

Therefore, the objective of this work was to produce a probiotic product to be used as an additive for animal feed. First, we studied the fed-batch fermentation of whey inoculated with a combination of kefir grains AGK1 and the free biomass present in fermented whole milk (Asturiana, Siero, Spain), previously used to activate the kefir grains. Control fermentations of whey inoculated only with kefir grains or fermented milk (Asturiana whole, semi-skim and skim milk) were also conducted to obtain data for the comparisons. All fed-batch fermentations were conducted at room temperature and 200 rpm, and the kinetics of the main culture variables (pH, free biomass, viable cells, lactose, glucose, galactose, proteins, lactic acid, acetic acid, glycerol, ethanol and antibacterial activity) were discussed. Second, some probiotic properties of the kefir cultures were analyzed, including their survival at low pH values, under simulated gastric and intestinal conditions, after freezing in skim milk at −20 °C, and in the commercial feed during storage at room temperature.

## 2. Results and Discussion

### 2.1. Kinetics of Whey Cultures Inoculated with Different Inocula

For the production of the probiotic product in whey, the fermentations were carried out at 200 rpm using inocula with a different microbiological composition (Table 1). This approach would allow obtaining information about the kinetics of the fermentation process, which will surely be influenced by the heterogeneous microbiota present in the kefir grains [16].

**Figure 1:**
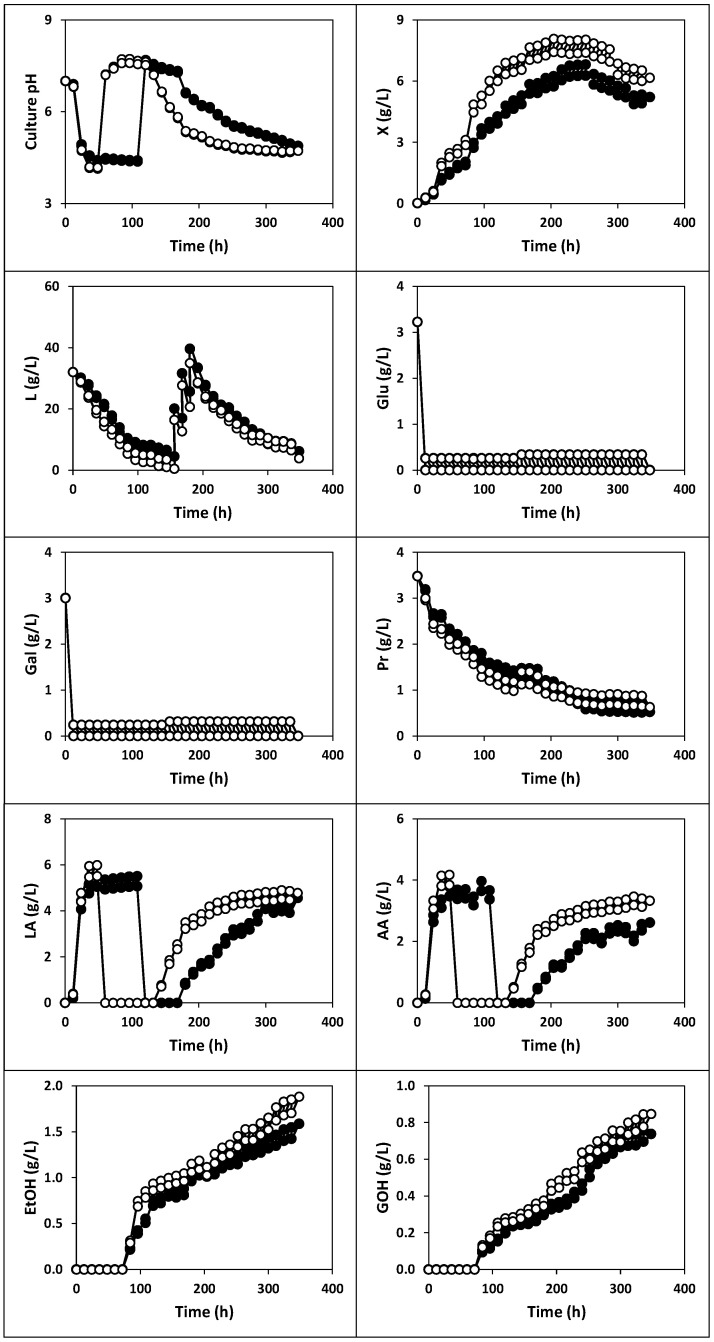
Fermentation kinetics (200 rpm) of whey with previously activated kefir grains AGK1 (1%, *w*/*v*) and fermented whole milk (1%, *v*/*v*) used in the activation (**white circles**), or with the previously activated kefir grains AGK1 (1%, *w*/*v*) (**black symbols**). X: biomass, L: lactose, Glu: glucose, Gal: galactose, LA: lactic acid, AA: acetic acid, Pr: proteins, EtOH: ethanol, GOH: glycerol. The experimental data represent the means of two cultures carried out in parallel with two analytical replicates each.

**Figure 2:**
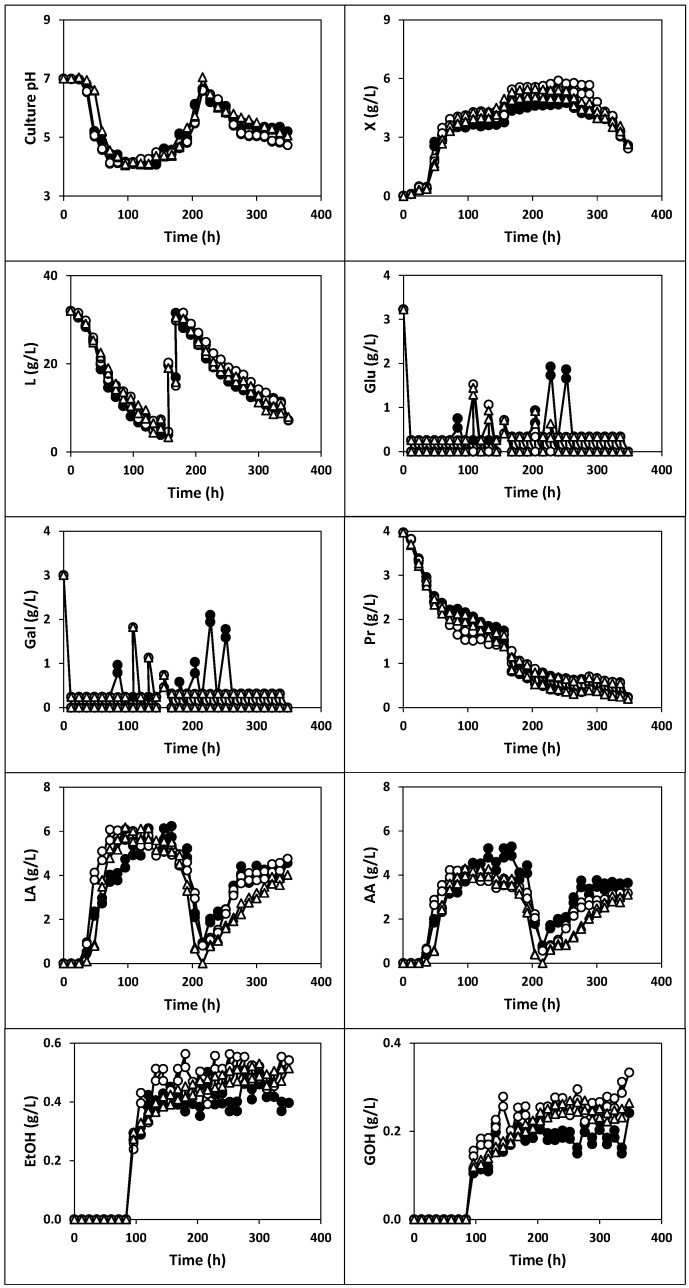
Fermentation kinetics (200 rpm) of whey with total free biomass from whole (**black circles**), semi-skim (**white circles**) and skim (**triangles**) milk previously fermented with kefir grains AGK1 with stirring at 150 rpm. X: biomass, L: lactose, Glu: glucose, Gal: galactose, LA: lactic acid, AA: acetic acid, Pr: proteins, EtOH: ethanol, GOH: glycerol. The experimental data represent the means of two cultures carried out in parallel with two analytical replicates each.

**Figure 3:**
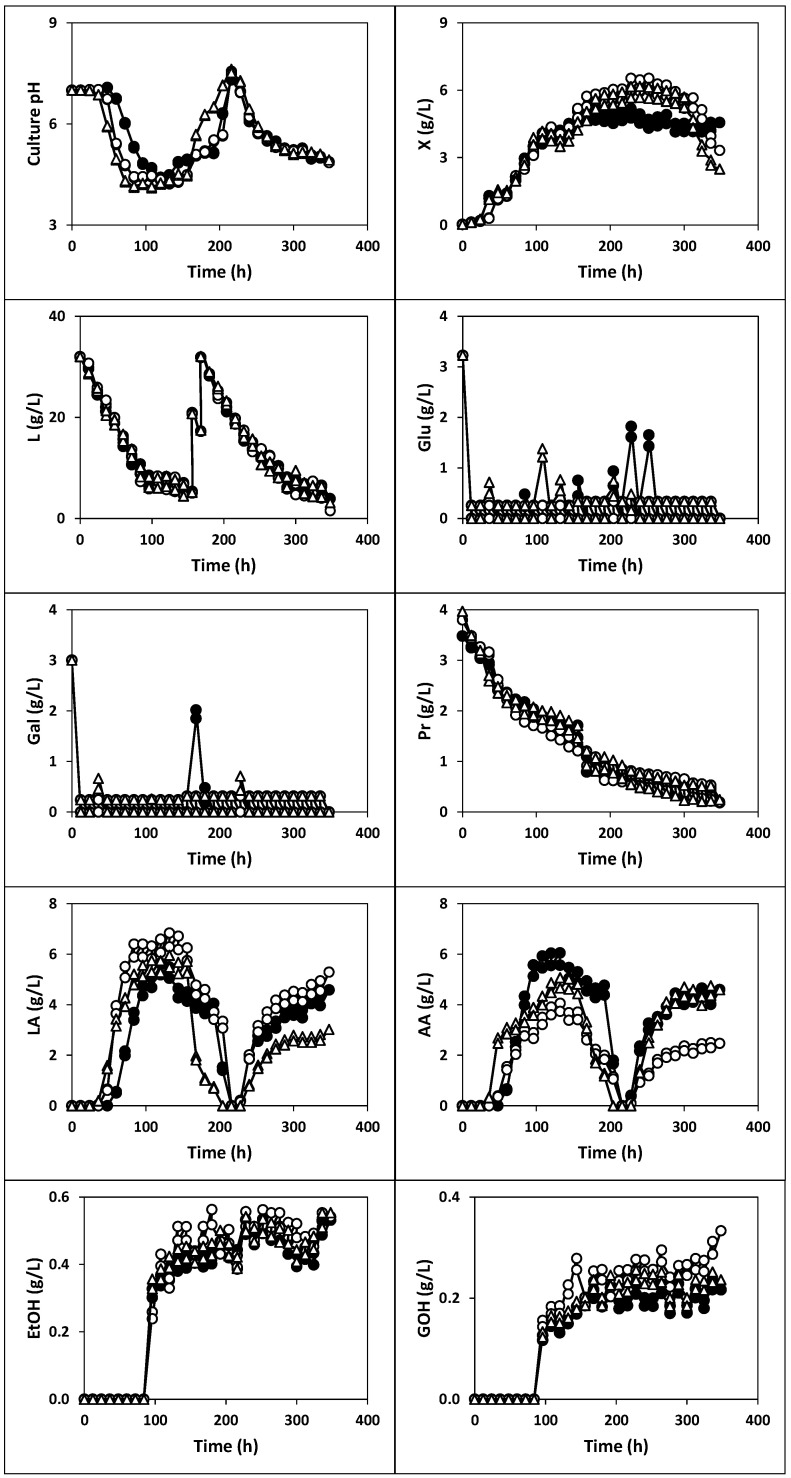
Fermentation kinetics (200 rpm) of whey with total free biomass from whole (**black circles**), semi-skim (**white circles**) and skim (**triangles**) milk previously fermented with kefir grains AGK1 without stirring. X: biomass, L: lactose, Glu: glucose, Gal: galactose, LA: lactic acid, AA: acetic acid, Pr: proteins, EtOH: ethanol, GOH: glycerol. The experimental data represent the means of two cultures carried out in parallel with two analytical replicates each.

**Figure 4:**
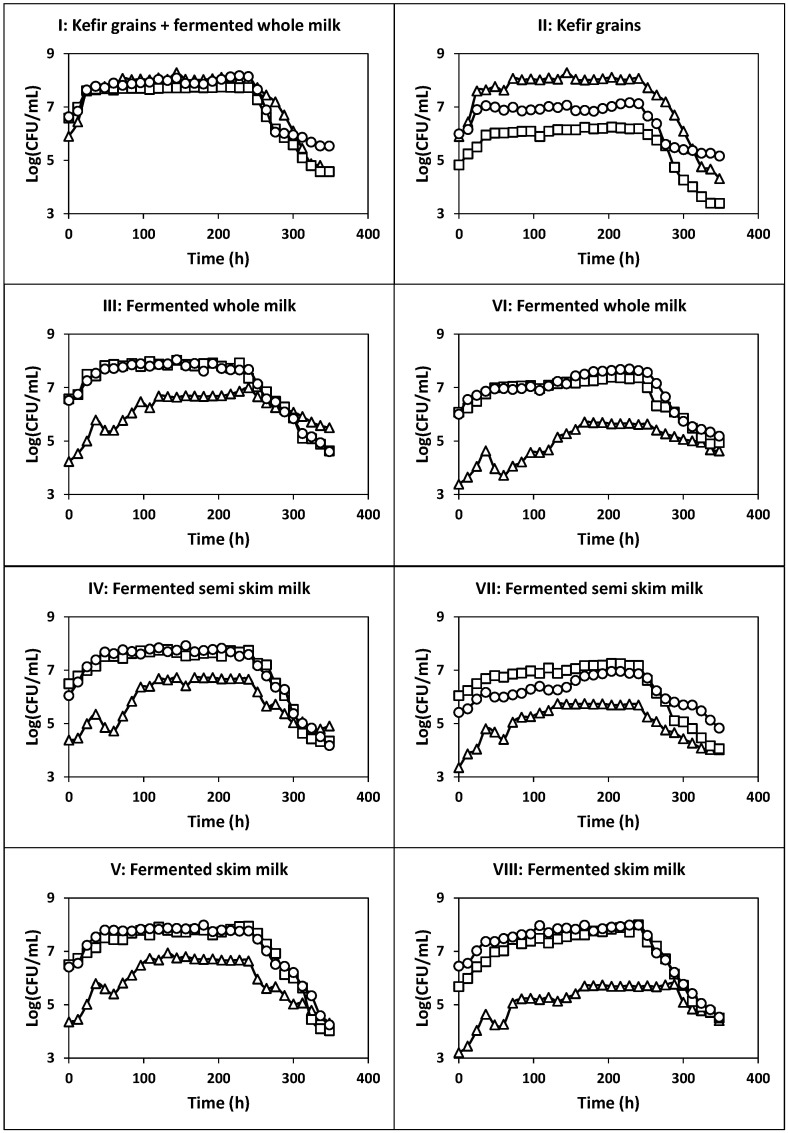
Kinetics of growth of viable LAB (**circles**), AAB (**squares**) and yeasts (**triangles**) in the different fed-batch fermentations in deproteinized whey. In fermentations III, IV and V, the culture medium was inoculated with milk fermented with kefir grains with agitation at 150 rpm. In fermentations VI, VII and VIII, the culture medium was inoculated with milk fermented with kefir grains under static conditions.

**Figure 5:**
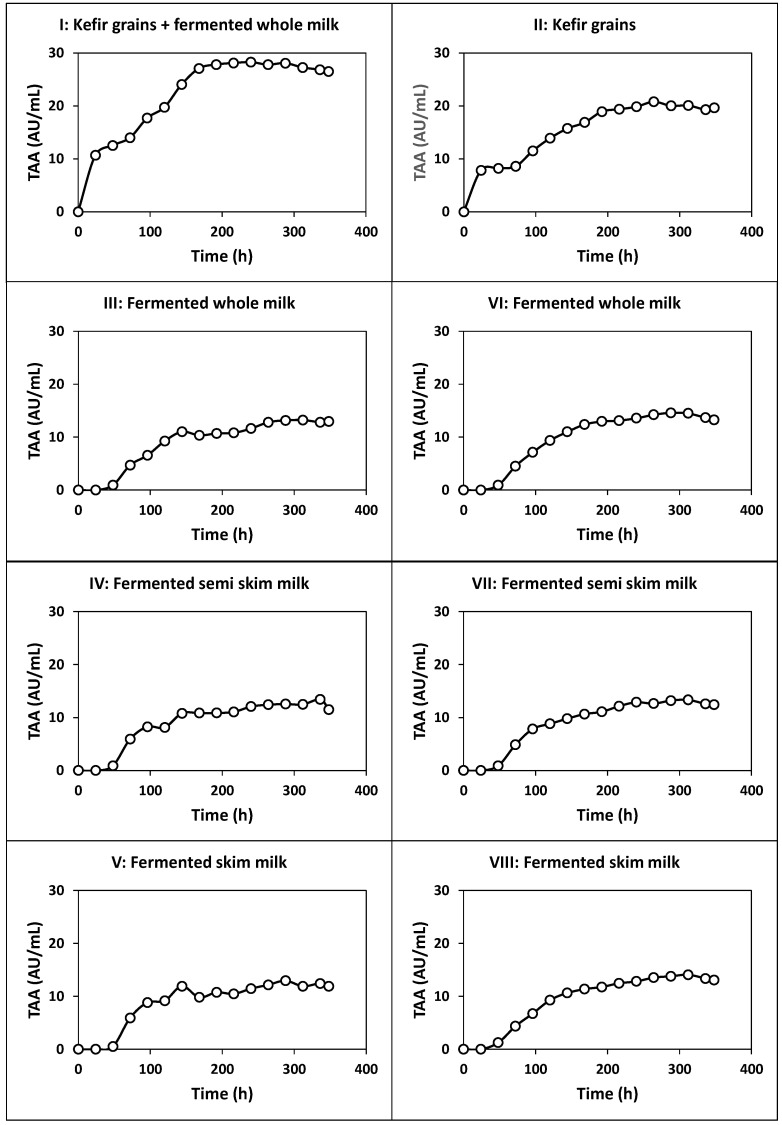
Kinetics of total antibacterial activity (TAA) production in the different fed-batch fermentations in deproteinized whey. In fermentations III, IV and V, the culture medium was inoculated with milk fermented with kefir grains with agitation at 150 rpm. In fermentations VI, VII and VIII, the culture medium was inoculated with milk fermented with kefir grains under static conditions.

The kinetics of culture pH, growth, nutrients consumption and products formation (Figure 1, Figure 2 and Figure 3), viable cell counts (Figure 4) and total antibacterial activity synthesis (Figure 5) in the different fed-batch cultures in whey are discussed below.

#### 2.1.1. Fermentation of Whey with Kefir Grains and Fermented Whole Milk (Fermentation I)

The results obtained in the fermentation of whey inoculated with 1% (*w*/*v*) of kefir grains plus 1% (*v*/*v*) of the fermented whole milk used in the activation of these kefir grains (fermentation I) are shown in Figure 1. As can be seen, the evolution of the culture variables: pH, free biomass, nutrients (lactose, glucose, galactose and proteins) and products (lactic and acetic acids, ethanol and glycerol) in this culture showed profiles different from those of fermentation with a pure culture [17,18].

The kinetics of the culture pH showed an unusual profile since it exhibited five well-differentiated phases. Thus, the usual pH drop during the first 48 h of incubation (from pH 7.00 to 4.14) was followed by an unusual abrupt increase from 48 to 60 h (until pH 7.22). Then, the culture pH increased slightly to 7.72 (60–96 h) but decreased linearly to 7.62 (96–120 h) and exponentially (120–348 h) until reaching a final value of 4.72 (Figure 1). These phases of pH decrease (0–48 h), increase (48–60 and 60–96 h) and decrease (96–120 and 120–348 h), paralleled the production of lactic and acetic acid, their consumption and new synthesis of both acids, respectively (Figure 1).

The realkalization of the culture medium observed between 48 and 96 h seems to be related to the consumption of organic acids (lactic and acetic) by the yeasts present in the microbiota of the kefir grains, which begins at 48 h in this culture. This consumption caused the total depletion of both organic acids observed between the 60 to 132 h of incubation (Figure 1). This hypothesis is supported by the results obtained by other researchers [19], who observed that *Candida guilliermondii* is capable of jointly assimilating acetic acid (at concentrations below 3.0 g/L) and xylitol so that this strain was considered as a detoxifying agent in the culture medium. More recently, Kirtadze and Nutsubidze [20] demonstrated the ability of *Saccharomyces cerevisiae* var. vini-39 to use acetic acid as a carbon source and that the presence of acetic acid in the medium contributes to the assimilation of lactic acid. In addition, the reduction in lactic acid levels seem to be associated with its assimilation by non-lactose consuming yeasts (e.g., *Torulaspora delbruekii* and *S*. *cerevisiae*), which are present in the kefir grains. This hypothesis is in perfect agreement with the results obtained by other researchers [21,22,23,24], who observed that in mixed cultures of *Lactobacillus kefiranofaciens* and *S. cerevisiae*, the lactic acid produced by the lactic bacterium was assimilated by yeast.

However, the release of amino acids or peptides that contain basic groups in their side chains, in addition to those present at the end of each molecule (all deprotonated at acidic pHs), could also contribute to the increase in pH to 7.72. In this way, it has been reported that lactic acid bacteria (LAB) can produce lactocepin (EC 3.4.21.96), a proteinase associated with the cell wall, which catalyzes the hydrolysis of caseins present in whey to produce amino acids [25]. The release of ammonium ion (NH4^+^) due to proteolysis of amino acids also contributes to increasing the culture pH [25].

Lactose concentration decreased almost exponentially to 0.99 g/L during the first 144 h of incubation when the growing culture was fed with fresh diluted whey during this period. Therefore, from 144 h of fermentation, the growing culture was fed with a lactose-supplemented concentrated whey (lactose, 200 g/L; proteins, 4.57 g/L; glucose, 5.04 g/L and galactose, 3.16 g/L) to restore the initial lactose concentration (34.98 g/L) in the fermentation substrate, that was reached at 180 h (Figure 1). From this time, the culture was fed again with fresh diluted whey to avoid the manifestation of the substrate inhibition phenomenon and accumulation of lactose in the culture medium. With this approach, the concentration of the carbon source decreased to a final value of 3.85 g/L.

The highest consumptions of lactose (19.66, 11.75 and 11.93 g/L) were observed during the first 48 h when the pH dropped from 7.00 to 4.16, from 156 to 180 h (pH was between 6.15 and 5.33) and from 180 to 204 h (pH was between 5.37 and 5.16). These pH values probably favored lactose consumption by LAB and AAB because nutrients assimilation in these species is higher at pH levels near 6.00 [2,26]. However, it is also probable that some lactose- (e.g., *S. unisporus*, *Candida tenuis* and *C. kefir*), glucose- and galactose-assimilating yeast species (*e.g., S. unisporus*, *C. tenuis*, *C. kefir*, *T. delbruekii* and *S. cerevisiae*) consumed lactose during fermentation. In this sense, it has recently been observed that the maximum specific velocity of *S. cerevisiae* T73 is favored at pH levels slightly higher than 4.80 [27]. This suggests that the pH levels (between 4.40 and 4.90) reached between 96 and 156 h of culture can facilitate the assimilation of lactose by yeasts and favor the growth of these microorganisms [28].

The initial amounts of glucose and galactose present in the whey and those supplied in each feeding were completely consumed, probably because of their low concentrations in the fermentation medium and rapid assimilation compared to lactose [2,29].

The free biomass production described a diauxic pattern with two cycles of growth (0–72 h and 72–252 h) in parallel with a biphasic consumption of proteins and the last phase of growth decline (Figure 1). In these growth phases, the biomass concentration increased to 3.11 (0–72 h) and 8.03 g/L (72–252 h) but then decreased to 6.16 g/L (252–348 h). This behavior has been observed before in realkalized fed-batch cultures with *Pediococcus acidilactici* NRRL B-5627 [30], *Lactococcus lactis* CECT 539 [17,18,29,31,32] and *Lactobacillus casei* CECT 4043 [2], which are species present in the kefir grains [33,34,35].

The growth slowdown observed after 252 h of fermentation cannot be associated with the depletion of the carbon source, since at that time, the lactose concentration in the culture medium after feeding was 15.11 g/L. Therefore, the carbon source could not be considered as a limiting substrate for the growth of the microbiota of the kefir grains.

The relatively low concentration of proteins in the fermentation medium (0.92 g/L) just after feeding and the low pH value (4.84 or lower) from 252 h could be possible causes to explain the decrease in biomass production. Thus, this low protein concentration could lead to a low concentration or depletion of some essential amino acid for the growth of the microbiota of the kefir grains, as observed before [30,36,37]. In addition, the slight protein consumption observed from 252 h to the end of the fermentation suggests that in this period, the remaining protein concentration in the culture medium could be a “harder” nitrogen source and consequently, more difficult to metabolize by the growing culture, which would, in fact, lead to a nutritional limitation [38,39]. In this period, the protein concentration decreased slightly after feeding, probably because of the consumption of the more easily assimilable protein fraction supplemented with the successive feeding with fresh whey.

On the other hand, low pH values could cause acidification of the cytoplasm of cells and collapse of the proton motive force, limiting the cytoplasmic processes [26,39] and nutrient transport [2,26].

Additionally, the depletion of some essential vitamins for the growth of bacteria or yeasts could also explain the slowdown and subsequent reduction in the biomass concentration [38,39].

Ethanol and glycerol were detected in the culture medium at 84 h of fermentation, and their concentrations increased in parallel with the increase in the biomass concentration in the second growth phase (Figure 1) and the growth of yeasts (Figure 4) until reaching final concentrations of 1.88 g ethanol/L and 0.85 g glycerol/L. The ethanol production obtained in fermentation I was considerably lower than those obtained by other researchers in the fermentation of whey with kefir grains: between 3.60 and 8.30 g/L [40], 13.41 g/L [13], between 13.41 and 26.98 g/L [14] and between 7.80 and 8.70 g/L [15].

The total count of viable cells (TCVC, as the sum of the counts of LAB, AAB and yeasts) similarly evolved and described a diauxic growth pattern (Figure 4), as observed for free biomass production (Figure 1). The maximum TCVC (3.6 × 10^8^ CFU/mL) was obtained at 144 h of fermentation, with LAB, AAB and yeast counts of 1.2 × 10^8^, 5.2 × 10^7^, and 1.9 × 10^8^ CFU/mL, respectively.

This is an important issue to be considered in the production of a probiotic product since it has been reported that a concentration of viable probiotic cells of at least 10^6^ CFU per mL or gram is needed to observe a beneficial physiological effect on farm animals [2,4,41].

The total antibacterial activity (TAA), which comprises the antibacterial activity of organic acids (lactic and acetic), alcohols (ethanol and glycerol) and bacteriocins, increased in parallel with the growth of free biomass (see Figure 1 and Figure 5), showing primary metabolite kinetics, as observed before for other antibacterial compounds, including nisin [36] and pediocin [30,42]. The maximum TAA (28.26 AU/mL) was obtained after 240 h of fermentation. The production of this antibacterial activity represents a competitive advantage that the microbiota of the kefir grains has over potential pathogens in colonizing the gastrointestinal tract, allowing the farm animals to grow healthily [2,43].

#### 2.1.2. Fermentation of Whey with Kefir Grains (Fermentation II)

After conducting the previous culture, the following experiment was focused on the fed-batch fermentation of whey with kefir grains. The results obtained are shown in Figure 1, Figure 4 and Figure 5.

From the comparison between the free biomass production in fermentations I and II, it could be noted that the highest concentrations were always obtained in the culture inoculated with kefir grains and fermented whole milk (Figure 1). This result is probably because, in the latter culture, the whey was inoculated with a higher concentration of biomass (those present in both the kefir grains and fermented milk) than in fermentation II. From a practical point of view, this observation indicates that it is necessary to inoculate the whey with both the kefir grains and fermented milk used for activation of the grains to obtain a highly concentrated probiotic preparation.

From a kinetic point of view, it can be noted that the biomass growth in fermentation II described a profile similar to that observed in the first culture (Figure 1): biomass concentration increased to 2.03 g/L (0–72 h) and 6.81 g/L (72–252 h) and then decreased to 5.21 g/L (252–348 h). This decline in biomass concentration started when the lactose and protein concentrations after feeding were 17.70 and 0.82 g/L, respectively.

Although the culture pH evolved, describing the same cycles observed during the first fermentation, the acidity levels in fermentation II showed some differences from those observed in fermentation I. Thus, in the second culture, the pH decreased from 7.00 to 4.38 at 48 h, when lactic and acetic levels increased to 5.48 and 3.78 g/L, respectively. The subsequent reduction in the concentrations of organic acids (from 108 to 168 h) to undetectable levels coincided with the increase in pH to values above 7.00 in this period (Figure 1). From 168 h of fermentation until the end of the incubation, the culture pH decreased logistically to a level of 4.88.

Regarding the carbon source, it can be observed that lactose was consumed exponentially during the first 156 h of cultivation from 32.00 to 4.45 g/L, in parallel with the increase in the production of biomass and organic acids. Then, the lactose concentration increased linearly until 39.49 g/L, between 156 and 180 h, because the feeding with lactose-supplemented concentrated whey and decreased exponentially again until the end of fermentation, reaching a level of 6.13 g/L. The consumption of glucose and galactose was similar to those observed in previous fermentation.

The proteins were more intensively consumed during the first 144 h of incubation. However, after this time, their consumption became softer, and protein concentrations decreased slightly to 0.98 g/L (108 h), probably due to the above-mentioned accumulation of the protein fraction non-assimilable by the microflora present in the kefir grains AGK1. Then, proteins concentration decreased exponentially to 0.52 g/L at the end of the incubation.

The production of ethanol and glycerol in the culture medium was detected at 96 h of fermentation, with their maximum concentrations (1.58 and 0.76 g/L) obtained at 348 and 336 h of fermentation, respectively.

In this culture, the maximum TCVC obtained at 144 h of fermentation (2.1 × 10^8^ CFU/mL) was also higher than 10^6^ CFU/mL. The viable cell counts for LAB, AAB and yeasts at this fermentation time were 1.2 × 10^7^, 1.4 × 10^6^ and 1.9 × 10^8^ CFU/mL, respectively.

The production of total antibacterial activity in fermentation II also exhibited primary metabolite kinetics as observed in the previous culture (see Figure 1 and Figure 5), but the maximum TAA (20.78 AU/mL obtained at 264 h of fermentation) was 26.47% lower than that obtained in fermentation I.

#### 2.1.3. Fermentation of Whey with Fermented Milk (Fermentations III, IV and V)

The first series of these cultures were conducted with deproteinized whey inoculated with 1% (*v*/*v*) of whole (fermentation III), semi-skim (fermentation IV) or skim (fermentation V) milk, previously fermented with kefir grains AGK1 with agitation at 150 rpm (Figure 2, Figure 4 and Figure 5).

In these fermentations, the culture variables described kinetics profiles similar to those observed in fermentations I and II, although the culture pH did not show the subsequent increase after abrupt realkalization of the fermentation medium observed in the previous cultures (Figure 1 and Figure 2).

In any case, the maximum production of free biomass and antibacterial activity in fermentations III (5.17 g/L and 13.21 AU/mL), IV (5.90 g/L and 13.45 AU/mL) and V (5.05 g/L and 12.94 AU/mL) were lower (*p* < 0.05) than those obtained in cultures I (8.07 g/L and 28.26 AU/mL) and II (6.81 g/L and 20.78 AU/mL).

Although fermentation I provided the highest total count of viable cells (3.6 × 10^8^ CFU/mL), the maximum TCVC in fermentations III, IV and V were also higher than 10^6^ CFU/mL. Thus, the maximum TCVC obtained in the latter cultures were as follows: 2.2 × 10^8^ CFU/mL in fermentation III at 144 h (LAB: 1.1 × 10^8^ CFU/mL, AAB: 1.1 × 10^8^ CFU/mL and yeasts: 4.4 × 10^6^ CFU/mL), 1.3 × 10^8^ CFU/mL in fermentation IV at 120 h (LAB: 6.9 × 10^7^ CFU/mL, AAB: 5.6 × 10^7^ CFU/mL and yeasts: 4.8 × 10^6^ CFU/mL) and 1.6 × 10^8^ CFU/mL in fermentation V at 180 h (LAB: 9.7 × 10^7^ CFU/mL, AAB: 6.3 × 10^7^ CFU/mL and yeasts: 5.1 × 10^6^ CFU/mL).

In fermentations III, IV and V, the lactose was almost completely consumed during the first 156 h of incubation until reaching remaining concentrations of 4.00, 4.64 and 3.32 g/L, respectively. After supplementing the growing culture with concentrated whey (at 156 and 168 h), the concentration of the carbon source decreased exponentially to 7.16, 7.29 and 7.95 g/L, respectively.

The last cultures were conducted by inoculating the deproteinized whey with 1% (*v*/*v*) of whole (fermentation VI), semi-skim (fermentation VII) or skim (fermentation VIII) milk, previously fermented with kefir grains under static conditions (Figure 3, Figure 4 and Figure 5). With this approach, it could determine whether agitation in the production of inoculum affects the kinetics of the cultures in whey.

From the comparison between fermentations III, IV, V, VI, VII and VIII (Figure 2 and Figure 3), it could be noted that only the maximum productions of free biomass, lactic acid and acetic acid were slightly higher in the fermentations inoculated with milk fermented without agitation, even though, the evolutions of the three variables did not show clear differences. However, both the kinetics and levels of the other culture variables (pH, lactose, glucose and galactose, proteins, ethanol, glycerol and antibacterial activity) were similar in the two series of fermentations.

The maximum production of free biomass and antibacterial activity in fermentations VI (5.18 g/L and 14.48 AU/mL), VII (6.53 g/L and 13.36 AU/mL) and VIII (5.68 g/L and 14.06 AU/mL) were comparable (*p* > 0.05) to those obtained in cultures III, IV and V (Figure 2, Figure 3 and Figure 5).

The maximum TCVC obtained in fermentations VI at 228 h (7.2 × 10^7^ CFU/mL: 5.0 × 10^7^ CFU LAB/mL, 2.2 × 10^7^ CFU AAB/mL and 4.6 × 10^5^ CFU yeasts/mL), VII at 204 h (2.7 × 10^7^ CFU/mL: 9.0 × 10^6^ CFU LAB/mL, 1.8 × 10^7^ CFU AAB/mL and 5.0 × 10^5^ CFU yeasts/mL) and VIII at 240 h (2.0 × 10^8^ CFU/mL: 9.7 × 10^7^ CFU LAB/mL, 1.0 × 10^8^ CFU AAB/mL and 4.8 × 10^5^ CFU yeasts/mL) were also higher than the threshold value of 10^6^ CFU per mL or gram needed to observe beneficial physiological effects in the host [41].

From a practical point of view, it could be considered that the inoculation of whey with fermented whole, semi-skim or skim milk is a good alternative to produce probiotic cultures with high viability to be used in animal feed. With this approach, a lower mass of kefir grains could be needed, and higher volumes of whey could be fermented to produce the probiotic product, with the additional advantage of reducing the environmental impact caused by the disposal of this food waste.

### 2.2. Evaluation of Some Probiotic Properties of the Kefir Cultures

After production of the concentrated probiotic cultures, the following experiments were focused on the evaluation of some of their probiotic properties, including their survival at low pH values, under simulated gastric and intestinal conditions, after freezing in skim milk at −20 °C and in the commercial feed during storage at room temperature. The results obtained are discussed below.

#### 2.2.1. Survival at Acidic pH Values

**Figure 6:**
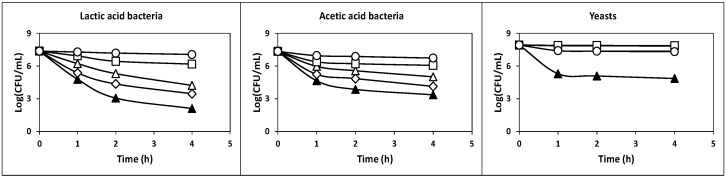
Survival of LAB, AAB and yeasts incubated in phosphate-buffered saline at pH values 1.0 (**black triangles**), 2.0 (**diamonds**), 3.0 (**white triangles**), 4.0 (**squares**) and 5.0 (**circles**). The experimental data represent the means of three experiments and two analytical replicates.

The results obtained in this assay are shown in Figure 6. As can be observed, when incubated at pH 1.0 for 4 h, initial LAB (7.37-log_10_) and AAB (7.35-log_10_) counts decreased considerably (*p* < 0.05) to 2.10-log_10_ and 3.37-log_10_, respectively. However, with the increase in incubation pH from 2.0 to 5.0, bacterial counts after 4 h of incubation were from 3.46-log_10_ to 7.06-log_10_ in the case of LAB, and from 4.14-log_10_ to 6.74-log_10_ in the case of AAB (Figure 6). In the case of yeasts, the initial counts (7.93-log_10_) decreased to final counts from 4.86-log_10_ to 7.87-log_10_ when the incubation pH increased from 1.0 to 4.0. However, the final counts of yeasts decreased to 7.32-log_10_ at pH 5.0. Therefore, the initial TCVC 1.3 × 10^8^ CFU/mL (as the sum of the counts of LAB, AAB and yeasts) decreased to 7.5 × 10^4^, 2.3 × 10^7^, 7.0 × 10^7^, 7.7 × 10^7^ and 3.8 × 10^7^ CFU/mL after 4 h of exposure to incubation pHs 1.0, 2.0, 3.0, 4.0 and 5.0, respectively.

#### 2.2.2. Survival under Simulated Gastric and Intestinal Transit

**Figure 7:**
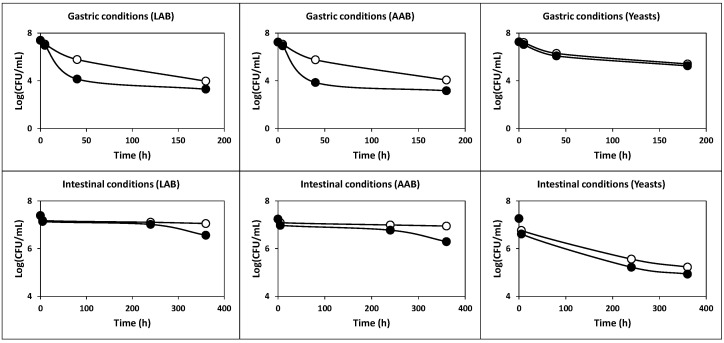
Survival of the populations of LAB, AAB and yeasts under simulated gastric (pH 2.0 + pepsin) and intestinal (pH 8.0 + pancreatin) transit (**black circles**). Controls (**white circles**) consisted of samples at the same pH values without enzymes. The experimental data represent the means of three experiments and two analytical replicates.

The results obtained in this assay (Figure 7) showed that the initial cell count for the three groups of microorganisms (LAB, AAB and yeasts) exhibited an exponential decrease during exposure to gastric conditions. Thus, after 180 min of incubation, the initial viable cell counts (6.97-log_10_ in the case of LAB, 7.24-log_10_ in the case of AAB and 7.26-log_10_ in the case of yeasts) declined by 4.09-log_10_, 4.08-log_10_ and 2.01-log_10_, respectively (*p* < 0.05). Therefore, the final cell counts were 2.0 × 10^3^, 1.5 × 10^3^ and 1.8 × 10^5^ CFU/mL, in the cases of LAB, AAB and yeasts, respectively. The TCVC after exposure to gastric conditions was 1.8 × 10^5^ CFU/mL.

However, after exposure to simulated intestinal conditions, the declines in viable cells for LAB, AAB and yeasts were smoother than those observed in the assay of tolerance to gastric conditions (Figure 7). Therefore, the final cell counts for LAB, AAB and yeasts after 360 min of incubation were 3.7 × 10^6^, 2.0 × 10^6^ and 8.6 × 10^4^ CFU/mL, respectively, being the final TCVC equal to 5.7 × 10^6^ CFU/mL.

These results indicated that strains of the milk kefir grains are intrinsically sensitive to gastric transit and intrinsically tolerant to intestinal transit, as observed before for *Lactococcus lactis* subsp. *lactis* CECT 539, *Pediococcus acidilactici* NRRL B-5627, *Lactobacillus casei* subsp. *casei* CECT 4043 and *Enterococcus faecium* CECT 410 [4].

#### 2.2.3. Survival after Freezing in Skim Milk at −20 °C

**Figure 8:**
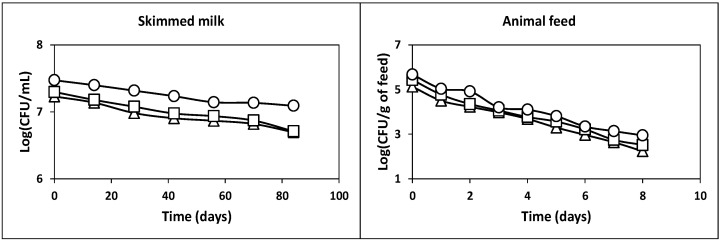
Survival of the populations of LAB (**circles**), AAB (**squares**) and yeasts (**triangles**) during storage at −20 °C with skim milk (**left** part) and in the commercial feed at room temperature (**right** part). The experimental data represent the means of three experiments and two analytical replicates.

After the production of probiotic cultures in whey, it is important to use an appropriate method to preserve them for a long time [4]. For this reason, the freezing method was used to study the survival of the probiotic culture obtained in this work (Figure 8) since this preservation procedure was more effective and less expensive than the freeze-drying method [4,44].

As observed in the left part of Figure 8, the viability of LAB, AAB and yeasts decreased slightly from 3.0 × 10^7^, 2.0 × 10^7^ and 1.7 × 10^7^ CFU/mL to 1.2 × 10^7^, 5.1 × 10^6^ and 5.0 × 10^6^ CFU/mL, respectively. Thus, the observed viability losses (0.38-log_10_, 0.59-log_10_ and 0.53-log_10_, in the case of LAB, AAB and yeasts) indicated that these microorganisms had good viability after 84 days of freezing storage (−20 °C).

#### 2.2.4. Survival in Commercial Piglet Feed at Room Temperature

A previous study showed that feed could be used as a vehicle to administer probiotic culture to animals [4]. For this purpose, high concentrations of viable cells of LAB, AAB and yeasts should be present in animal feed. Therefore, the following assay was focused on the study of the survival of the three microbial groups in the animal feed.

The results obtained are shown in the right part of Figure 8. As can be observed, the initial counts of LAB, AAB and yeasts decreased at average rates of 0.33-, 0.34- and 0.34-log_10_, respectively. Thus, the initial counts of viable cells of LAB, AAB and yeasts in the feed (4.7 × 10^5^, 2.7 × 10^5^ and 1.3 × 10^5^ CFU/g of feed, respectively) decreased to 8.9 × 10^2^, 3.3 × 10^2^ and 1.7 × 10^2^ CFU/g of feed, respectively, after 8 days of incubation. Therefore, the initial TCVC (8.8 × 10^5^ CFU/g of feed) decreased to a final value of 1.4 × 10^3^ CFU/g of feed, which is considerably lower than 10^6^ CFU/g of feed, the above-mentioned threshold value needed to observe beneficial physiological effects in the host [41]. This observation suggests that the animal feed should be supplemented daily with the probiotic culture to feed the animals with appropriate viable probiotic cell counts.

#### 2.2.5. Survival of Cells Protected with Skim Milk and Piglet Feed at Low pH Values and Simulated Gastrointestinal Conditions

**Figure 9:**
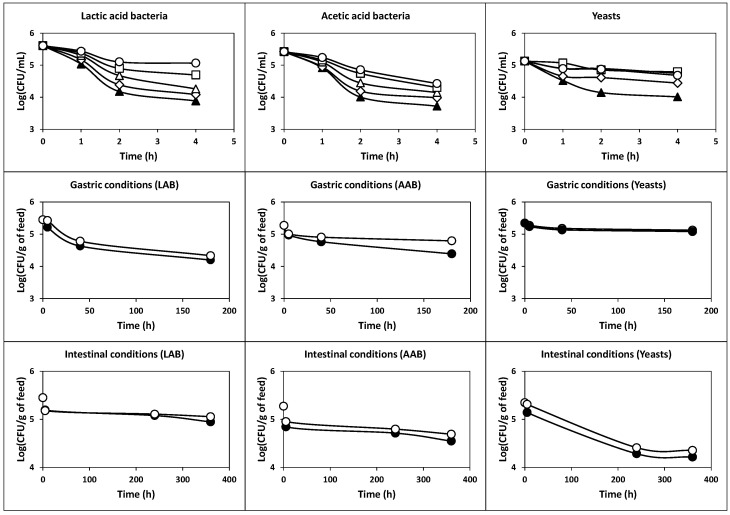
Survival of the populations of LAB, AAB and yeasts protected with skim milk and piglet feed at pH values 1.0 (**black triangles**), 2.0 (**diamonds**), 3.0 (**white triangles**), 4.0 (**squares**) and 5.0 (**circles**) and under simulated gastric (pH 2.0 + pepsin) and intestinal (pH 8.0 + pancreatin) transit (**black circles**). Controls (**white circles**) consisted of samples at the same pH values without enzymes. The experimental data represent the means of three experiments and two analytical replicates.

The results obtained in this study (Figure 9) showed a considerable increase in both the acid and gastrointestinal transit tolerance of the three groups of microorganisms (LAB, AAB and yeasts) compared to the corresponding tolerances of the washed cells observed in the assays without skim milk and piglet feed (Figure 6 and Figure 7). This important finding suggests that a higher number of probiotic microorganisms could reach and colonize the intestine [4].

Concerning the acidic pH tolerance, it could be noted that the initial LAB (5.61-log_10_) and AAB (5.42-log_10_) counts decreased to 3.88-log_10_ and 3.72-log_10_, respectively (*p* < 0.05) after incubation at pH 1.0 for 4 h. The counts of LAB and AAB at pHs from 2.0 to 5.0 after 4 h of incubation increased from 4.08-log_10_ to 5.06-log_10_, and from 3.98-log_10_ to 4.43-log_10_, respectively (Figure 9). The final counts of yeasts decreased from 5.13-log_10_ to 4.01-, 4.44-, 4.75-, 4.79- and 4.68-log_10_ at incubation pHs of 1.0, 2.0, 3.0, 4.0 and 5.0, respectively. Then, the initial TCVC of 8.0 × 10^5^ CFU/g in the piglet feed decreased to 2.3 × 10^4^, 4.9 × 10^4^, 8.8 × 10^4^, 1.3 × 10^5^ and 1.9 × 10^5^ CFU/g, after 4 h of exposure to incubation pHs of 1.0, 2.0, 3.0, 4.0 and 5.0, respectively.

With regard to gastrointestinal tolerance, it could be noted that after 180 min of incubation with simulated gastric juice, the initial viable cell counts (5.45-log_10_ in the case of LAB, 5.27-log_10_ in the case of AAB and 5.35-log_10_ in the case of yeasts) declined by 1.25-, 0.88- and 0.27-log_10_, respectively (*P* < 0.05). Thus, the final cell counts were 1.6 × 10^4^, 2.5 × 10^4^ and 1.2 × 10^5^ CFU/g, in the case of LAB, AAB and yeasts, respectively. The TCVC after exposure to gastric conditions was 1.6 × 10^5^ CFU/g, which is slightly lower than 10^6^ CFU/g.

In the case of simulated intestinal juice, the initial viable cell counts decreased by 0.50-log_10_, 0.73-log_10_ and 1.13-log_10_, in the case of LAB, AAB and yeasts, respectively (*P* < 0.05), after 360 min. Thus, the final cell counts were 8.9 × 10^4^, 3.5 × 10^4^, 1.6 × 10^4^ and 1.4 × 10^5^ CFU/g for LAB, AAB, yeasts and TCVC, respectively.

## 3. Materials and Methods

### 3.1. Inoculum Preparation, Culture Media and Fermentation Conditions

The milk kefir grains CIDCA AGK1 used in this study, kindly provided by the Center for Research and Development in Food Cryotechnology (CIDCA, La Plata, Argentina), were first activated with three transfers in pasteurized ultra-high temperature (UHT) whole milk (Central Lechera Asturiana, Asturias, Spain). The activation substrate (pH 6.75) contained (per liter): 46.0 g carbohydrates, 32.0 g proteins, 36.0 g fats, 24.0 g saturated fats, 1.0 g salt and 1.2 g calcium.

Activations were carried out at room temperature away from direct sunlight in Pyrex bottles containing pasteurized whole milk UHT (approximately 30.6 g of kefir grains per L of milk). The bottles were covered with a cheesecloth that was secured with a rubber band and incubated at 150 rpm for 24 h. After three activation cycles, the kefir grains were separated from the fermented milk under sterile conditions by filtration through a plastic strainer, and then kept at 4 and −20 °C in pasteurized whole milk UHT for storage at short and long times, respectively.

The mean composition (g/L) of diluted whey used as culture and feeding medium in the different fed-batch fermentations was: lactose, 32.06; total nitrogen, 0.51; total phosphorous, 0.29; soluble proteins, 3.48; and pH 7.00. The mean composition (g/L) of the concentrated whey used as feeding medium in the different fed-batch fermentations was: lactose, 50.00; total nitrogen, 0.96; total phosphorous, 0.39; soluble proteins, 4.57; and pH 7.00. The culture media were sterilized at 121 °C for 15 min [38,39].

In this work, the fermentation medium (diluted whey) was inoculated using four inoculation strategies. In the first one, the UHT whole milk was inoculated with the previously activated milk kefir grains CIDCA AGK1 (1%, *w*/*v*) and incubated in Pyrex bottles at room temperature at 150 rpm for 24 h. Both the kefir grains (1%, *w*/*v*) and the fermented whole milk (1%, *v*/*v*) were used as the inoculum in fermentation I. In the second strategy, only the kefir grains (1%, *w*/*v*) were used as inoculum in fermentation II.

In the third strategy, three types of substrates (UHT whole, semi-skim and skim milk) were inoculated with the previously activated milk kefir grains CIDCA AGK1 (1%, *w*/*v*) and incubated in Pyrex bottles at room temperature at 150 rpm for 24 h. Then, the culture medium (diluted whey) in fermentations III, IV and V was inoculated with 1% (*w*/*v*) of these fermented whole, semi-skim and skim milk, respectively.

In the fourth strategy, the three types of milk (UHT whole, semi-skim and skim) were inoculated with the previously activated milk kefir grains CIDCA AGK1 (1%, *w*/*v*) and incubated in Pyrex bottles at room temperature without agitation for 24 h. Then, the culture medium (diluted whey) in fermentations VI, VII and VIII were inoculated with 1% (*w*/*v*) of these fermented whole, semi-skim and skim milk, respectively.

All fermentations were carried out in duplicate at 200 rpm in Pyrex bottles containing 50 mL of diluted whey.

In these cultures, three strategies of feeding were used to feed the fed-batch cultures. First, the growing cultures were fed fresh diluted whey medium each 12 h, during the first 144 h of incubation. After this, the culture was fed a concentrated whey supplemented with lactose up to a concentration of 200 g/L until reaching almost the initial lactose concentration (32.0 g/L) in the fermentation medium (diluted whey). Subsequently, the growing culture was fed fresh diluted whey medium each 12 h, from 228 h until the end of the incubation. With this approach, the manifestation of the substrate inhibition phenomena due to the presence of high concentrations of lactose in the medium was avoided.

### 3.2. Analytical Determinations

The concentrations of biomass, sugars (lactose, glucose and galactose), lactic acid, acetic acid, ethanol, glycerol and proteins in each culture were determined in duplicate by methods described in a previous work [29].

### 3.3. Antibacterial Activity Quantification

The antibacterial activity against *Enterococcus faecium* CECT 410 (indicator strain) in each culture was quantified in duplicate using a photometric bioassay and expressed as activity units (AU) per milliliter cell-free supernatant, as described in Costas et al. [29].

*Enterococcus faecium* CECT 410 obtained from the Spanish Type Culture Collection (CECT) was used as the target organism in the antibacterial-activity assay. Working cultures of this strain previously maintained at 4 °C on Rothe agar were grown in Rothe broth at 200 rpm and 30 °C for 12 h.

### 3.4. Determination of Total Viable Counts

Serial decimal dilutions of samples from the cultures were made in sterile PBS and plated in triplicate in Petri dishes containing MRS (de Man, Rogosa and Sharpe) agar for enumeration of lactic acid bacteria (LAB) [45], Carr agar for enumeration of acetic acid bacteria (AAB) [46] and YEG (yeast extract-glucose) agar for enumeration of yeasts.

After sterilization, both the MRS and Carr agar were supplemented with amphotericin B up to a final concentration of 0.1 g/L to avoid fungal growth. In the same way, the YEG agar was supplemented with chloramphenicol up to a final concentration of 0.1 g/L to avoid bacterial growth.

The Petri dishes were incubated at 30 °C for 48 h (MRS and Carr agar) or 25 °C for 48 h (YEG agar). Then, the colonies on each agar medium were counted, and the results (means ± standard deviations of two experiments (two cultures carried out in parallel) and three analytical replications each) were expressed as log CFU (colony forming units) per mL fermented whey.

This procedure was also used to determine the viable counts of LAB, AAB and yeast strains after exposure to acidic pH, simulated gastric and pancreatic juices and during freezing storage (−20 °C) with skim milk and in the animal feed at room temperature.

### 3.5. Tolerance to Acidic pH

To obtain viable cells of LAB, AAB and yeasts, a fed-batch fermentation of diluted whey medium with kefir grains (1%, *w*/*v*) and fermented whole milk (1%, *v*/*v*) was carried out in duplicate at 30 ºC for 36 h. After separating the kefir grains by filtration with a sterile plastic strainer, the fermented medium (3 L) was centrifuged at 5000× *g* for 10 min at 4 °C. Subsequently, the pellets were washed in sterile phosphate-buffered saline (PBS: 10 mM sodium phosphate monobasic, 10 mM sodium phosphate dibasic, 130 mM sodium chloride, pH 7.2), and resuspended in 3 L sterile PBS [4]. This suspension of cells was diluted 1/100 in sterile PBS at pH 1.0, 2.0, 3.0, 4.0 and 5.0 for 1, 2 and 4 h in culture tubes in triplicate. After each incubation time, each suspension of cells was serially diluted (10-fold) in sterile PBS, and duplicate aliquots (0.1 mL) from these dilutions were then spotted on MRS, Carr or YEG agar plates for determining the number of surviving cells [4]. The initial viable cell counts for LAB, AAB and yeasts were determined as described above prior to assay of tolerance to acidic pH.

### 3.6. Preparation of Simulated Gastric and Pancreatic Juices

Gastric and pancreatic juices were prepared as described by Charteris et al. [47]. For the preparation of gastric juice, 3 g pepsin (Sigma-Aldrich Co. LLC, St. Louis, MO, USA) from the stomach mucosa and 5 g of NaCl were dissolved in sterile distilled water, and the mixture was adjusted to pH 2.0 with 12 M HCl. For the preparation of pancreatic juice, 1 g pancreatin (Sigma) from the porcine pancreas and 5 g of NaCl were dissolved in sterile distilled water, and subsequently, the enzymatic preparation was adjusted to pH 8.0 with 0.1 M NaOH.

### 3.7. Tolerance to Simulated Gastrointestinal Juices

The assay of the tolerance of LAB, AAB and yeasts to simulated gastrointestinal juices was conducted as described by Guerra et al. [4]. Thus, the fermented diluted whey (3 L) obtained after 36 h of fermentation (as described above in Section 3.5) was centrifuged at 5000× *g* for 10 min at 4 °C and washed in sterile PBS. The washed cells were resuspended in 3 L PBS [4]. Subsequently, triplicate samples (0.2 mL) of the resuspended cells were mixed with 1 mL of gastric or intestinal juice, and the mixtures were incubated at 37 ºC for 5, 40 and 180 min (for the assay of gastric transit tolerance) or 5, 240 and 360 min (for the assay of small intestinal transit tolerance). After each incubation time, duplicate aliquots of 0.1 mL were taken to determine the remaining viability of the resuspended cells of LAB, AAB and yeasts, as described above. The viable counts of the resuspended cell suspensions were also determined before the determination of gastric and intestinal transit tolerances [4].

### 3.8. Survival of the Microorganisms from Kefir Grains during Freezing Storage

For the preparation of the probiotic supplemented piglet feed, the fermented diluted whey obtained after 36 h of fed-batch fermentation was mixed with skim milk (300 g skim milk/L of fermented medium) and stored frozen at −20 °C in screw cap glass tubes [4].

The viability of these cultures was checked for 84 days. For this purpose, three tubes were taken each 14 days and defrosted at room temperature. Subsequently, serial 10-fold dilutions were made in sterile PBS, plated in duplicate on MRS, Carr or YEG agar in Petri dishes for LAB, AAB and yeasts enumeration, respectively. The Petri dishes were incubated at the corresponding temperatures for each microbial group for 84 days.

### 3.9. Survival of Microorganisms from Kefir Grains in the Piglet Feed stored at Room Temperature

In this assay, 20 mL of the culture with skim milk (300 g skim milk/L of fermented medium) and 1 kg of commercial piglet feed (its composition is given in Guerra et al. [4]) were mixed and stored at room temperature. Each 24 h, triplicate samples (10 g) of probiotic supplemented piglet feed were mixed 1:10 with sterile PBS and vortexed for 2 min, and then the samples were serially diluted using sterile PBS [4]. Subsequently, the different dilutions were plated in duplicate in MRS, Carr or YEG agar in Petri dishes for the enumeration of colonies of LAB, AAB and yeast, respectively. The Petri dishes were incubated at the corresponding incubation temperature for each microbial group for 8 days. The results were expressed as the number of colonies counted per gram (wet weight) of feed.

### 3.10. Effects of Skim Milk and Feed on the Tolerance of Cells to Acidic pH and Gastrointestinal Conditions

To determine whether the skim milk and piglet feed protect the LAB, AAB and yeast cells and increase their tolerance to acidic pH and gastrointestinal conditions, the fermented diluted whey obtained after 36 h of fed-batch fermentation was mixed with skim milk (30%, *w*/*v*) and then added to the piglet feed (2%, *v*/*w*). The mixture was then submitted to the in vitro tests of tolerance to acidic pH values and gastrointestinal conditions as described previously in Section 3.5 and Section 3.7.

### 3.11. Statistical Analysis

The culture pH, the concentrations of biomass, sugars (lactose, glucose and galactose), lactic acid, acetic acid, ethanol, glycerol, proteins and antibacterial activity and the counts (previously transformed by logarithm (log_10_)) of LAB, AAB and yeasts in the different fermentations were statistically analyzed using the paired samples *t*-test included in the software package IBM SPSS Statistics for Windows (Version 21.0, Armonk, NY, USA: IBM Corp., 2012). In each case, the difference was considered statistically significant when a *p*-value < 0.05 was obtained. The experimental values of culture pH and concentrations of biomass, sugars, organic acids, alcohols, proteins, antibacterial activity and viable cells were obtained in duplicate analytical assays from two cultures carried out in parallel. Therefore, the total number of experimental data for each variable in each experimental point was 2 (runs: fermentations) × 2 (replicates by analytical assay) = 4 total experimental data. The microbial counts in the assays of tolerance to acidic pH values, simulated gastrointestinal juices and during freezing storage (−20 °C) with skim milk and in the animal feed at room temperature were obtained from two cultures carried out in parallel, triplicate survival assays and duplicate counts. Therefore, in this case, the total number of experimental data for the counts of LAB, AAB and yeasts in each experimental point was 2 (runs: fermentations) × 3 survival assays × 2 (replicates: counts) = 12 total experimental data.

## 4. Conclusions

The results obtained in the present work indicate the feasibility of producing a concentrated probiotic culture with high viable cell counts of LAB, AAB and yeasts, by the fermentation of whey with the microbiota of kefir grains, with potential application as an additive in animal feed.

The experimental procedure used allowed obtaining important information about the kinetics of the fermentation process, determining the complex relationship between the culture variables and selecting the appropriate fermentation procedure to produce a probiotic culture with a high concentration of viable cells.

Fermentation of whey with 1% (*w*/*v*) of kefir grains plus 1% (*v*/*v*) of the fermented whole milk used in the activation of these kefir grains (fermentation I) provided a product with the highest concentrations of free biomass (8.03 g/L), total antibacterial activity (28.26 AU/mL) and total counts of viable cells (3.6 × 10^8^ CFU/mL, with LAB, AAB and yeasts counts of 1.2 × 10^8^, 5.2 × 10^7^ and 1.9 × 10^8^ CFU/mL, respectively). The latter viable counts were higher than the viable probiotic cell count (10^6^ CFU per mL or gram) needed to observe a beneficial physiological effect on farm animals. In addition, this probiotic product contained low concentrations of alcohols (1.88 g ethanol/L and 0.85 g glycerol/L).

After exposure of viable cells to acidic pH values for 4 h, a reduction of about one (at pH values between 2.0 and 5.0) or four (at pH 1.0) orders of magnitude was achieved. In the same way, the initial total counts of viable cells decreased in two or one order of magnitude after exposure to gastric (180 min) and intestinal (360 min) conditions.

The results obtained indicated that the viable probiotic strains protected with skim milk can be appropriately preserved for a long time by freezing storage (−20 °C). The addition of the probiotic culture to the animal feed decreased cell viability after 8 days of incubation, indicating that the probiotic animal feed should be prepared daily to feed the animals with appropriate viable probiotic cell counts. With this approach and considering that the protection of viable cells with skim milk and feed increased their tolerance to acidic pH and gastrointestinal conditions, compared with washed cells, a higher number of cells could reach and colonize the intestine of the host. This fact could produce positive effects on the health of the animals.

## Figures and Tables

**Table 1:** Microbiological composition (in mean colony forming units (CFU) ± standard deviation/mL or g) of the different inocula used in this work.

Inoculum	LAB (CFU/mL)	AAB (CFU/mL)	Yeasts (CFU/mL)
Kefir grains AGK1	9.9 × 10^7^ ± 1.3 × 10^7^ *	6.8 × 10^6^ ± 1.0 × 10^6^ *	7.8 × 10^7^ ± 1.6 × 10^7^ *
Fermented whole milk at 150 rpm	3.3 × 10^8^ ± 2.5 × 1.0^7^	3.8 × 10^8^ ± 2.2 × 1.0^7^	1.7 × 10^6^ ± 5.7 × 1.0^4^
Fermented semi-skim milk at 150 rpm	1.1 × 10^8^ ± 1.4 × 1.0^7^	3.1 × 10^8^ ± 7.1 × 1.0^7^	2.4 × 10^6^ ± 2.5 × 1.0^5^
Fermented skim milk at 150 rpm	2.6 × 10^8^ ± 4.4 × 1.0^7^	3.2 × 10^8^ ± 2.8 × 1.0^7^	2.3 × 10^6^ ± 2.0 × 1.0^5^
Fermented whole milk without agitation	1.0 × 10^8^ ± 7.8 × 1.0^6^	1.2 × 10^8^ ± 2.7 × 1.0^7^	2.4 × 10^5^ ± 5.0 × 1.0^4^
Fermented semi-skim milk without agitation	2.6 × 10^7^ ± 1.1 × 1.0^7^	1.1 × 10^8^ ± 5.3 × 1.0^6^	2.2 × 10^5^ ± 7.8 × 1.0^4^
Fermented skim milk without agitation	2.8 × 10^8^ ± 9.9 × 1.0^6^	4.8 × 10^7^ ± 1.4 × 1.0^6^	1.6 × 10^5^ ± 5.4 × 1.0^4^

* In CFU per g of kefir grains.

## Data Availability

Data are available in the manuscript.
